# Exploring the quantum critical behaviour in a driven Tavis–Cummings circuit

**DOI:** 10.1038/ncomms8111

**Published:** 2015-05-14

**Authors:** M. Feng, Y.P. Zhong, T. Liu, L.L. Yan, W.L. Yang, J. Twamley, H. Wang

**Affiliations:** 1 State Key Laboratory of Magnetic Resonance and Atomic and Molecular Physics, Wuhan Institute of Physics and Mathematics, Chinese Academy of Sciences, Wuhan 430071, China; 2 Department of Physics, Zhejiang University, Hangzhou 310027, China; 3 The School of Science, Southwest University of Science and Technology, Mianyang 621010, China; 4 Department of Physics and Astronomy, ARC Centre for Engineered Quantum Systems, Macquarie University, Sydney, New South Wales 2109, Australia; 5 Synergetic Innovation Center of Quantum Information and Quantum Physics, University of Science and Technology of China, Hefei, Anhui 230026, China

## Abstract

Quantum phase transitions play an important role in many-body systems and have been a research focus in conventional condensed-matter physics over the past few decades. Artificial atoms, such as superconducting qubits that can be individually manipulated, provide a new paradigm of realising and exploring quantum phase transitions by engineering an on-chip quantum simulator. Here we demonstrate experimentally the quantum critical behaviour in a highly controllable superconducting circuit, consisting of four qubits coupled to a common resonator mode. By off-resonantly driving the system to renormalize the critical spin-field coupling strength, we have observed a four-qubit nonequilibrium quantum phase transition in a dynamical manner; that is, we sweep the critical coupling strength over time and monitor the four-qubit scaled moments for a signature of a structural change of the system's eigenstates. Our observation of the nonequilibrium quantum phase transition, which is in good agreement with the driven Tavis–Cummings theory under decoherence, offers new experimental approaches towards exploring quantum phase transition-related science, such as scaling behaviours, parity breaking and long-range quantum correlations.

## Abstract (web summary)

Quantum phase transitions are a discontinuous change in a property of the ground state or the structure of the excited states as a system parameter traverses a critical point. Here, the authors recreate analogous effects with laboratory-achievable light-matter coupling in an on-chip superconducting circuit

## Graphical abstract

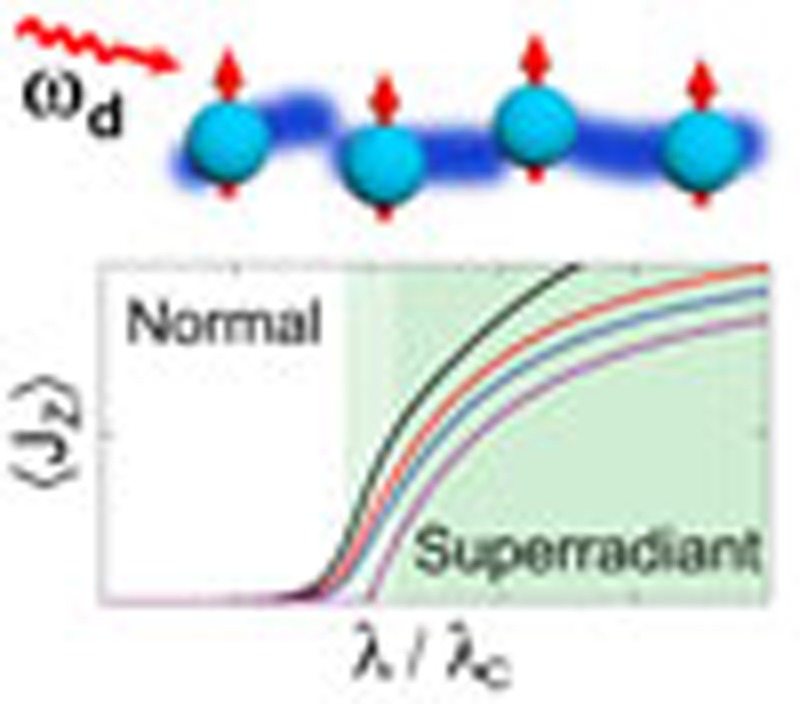

In a quantum phase transition (QPT)^1^,^2^,^3^,^4^,^5^,^6^,^7^,^8^, the quantum system displays nonanalytic behaviour, which is reflected by a discontinuous change in a property of the ground state or the structure of the excited states, when a system parameter traverses a critical point. In many cases this discontinuous change has a cusp-like character, surrounding which quantum fluctuations dominate and novel phenomena can be explored. QPTs are studied in a variety of naturally grown condensed matter materials such as conductors, superconductors and magnets. With the introduction of well-controlled quantum elements, ranging from cold atoms, photons and trapped ions to Josephson-junction qubits, it becomes possible to engineer a quantum simulator, an ordered arrangement of the above-mentioned quantum elements, to mimic and investigate the properties of complex interacting quantum materials. Achieving a QPT using fine-tuning knobs available in an experimentally accessible Hamiltonian presents the first step towards engineering such a simulator for exploring QPT-related physics in few or many-body interacting quantum systems.

Recently, there has been extensive interest to investigate a QPT in the Dicke model^9^ using artificially engineered systems both experimentally^10^ and theoretically^11^,^12^. As another paradigm to investigate light–matter interactions, the Tavis–Cummings (TC) model^13^ is an integrable variant of the Dicke model, which also yields significant interests covering a wide range of configurations such as the multimode resonator^14^ and the TC lattice^15^. The TC model is derived from the Dicke model in the rotating-wave approximation, which is valid when the spin-field coupling is weak in comparison with other characteristic frequencies of the system. It is generally understood that a QPT can occur in the Dicke model, rather than in the TC model, with the former critical spin-field coupling required to be equal to geometric mean of the spin and field resonance frequencies. However, most laboratory-achievable spin-field couplings can only reach the strengths that are many orders smaller than the Dicke critical coupling strength. Even for some systems with ultrastrong couplings^16^,^17^, the Dicke coupling strength is still unreachable. As a result, achieving the Dicke QPT with current laboratory techniques requires additional assistance. For example, it was shown that an external drive in a cold atom system leads to the Dicke Hamiltonian in the rotating frame, yielding a nonequilibrium QPT^10^.

Since most artificially engineered quantum systems can only reach coupling strengths that are within the TC model^18^,^19^,^20^, it would be of significant interest to see whether a nonequilibrium QPT can be observed within a driven TC model^21^,^22^. In comparison with the Dicke model under a drive, no approximation is necessary to transfer the driven TC model from the laboratory frame to the rotating frame. Within the rotating frame, a QPT is indeed predicated^22^ at a critical coupling strength below the Dicke critical coupling strength. This driven TC QPT critical coupling is comparable to the geometric mean of the spin and field detunings from the drive frequency (here and below referred to as the TC critical coupling, in comparison with the Dicke critical coupling).

Here we show experimental evidence confirming the existence of such a nonequilibrium QPT in a driven TC circuit, with four superconducting phase qubits each coupled, at a fixed strength smaller than the Dicke critical coupling by 200 times, to a superconducting coplanar waveguide resonator. We witness the nonequilibrium QPT through a dynamical measurement, by recording the time evolution of the four-spin joint occupation probabilities, while the TC critical coupling strength is swept over time. In the experiment we demonstrate the high level of control possible in our system by off-resonantly driving the common resonator mode and subsequently fine-tuning the qubit frequency to cross the TC QPT critical point, with the results measured at different microwave drive strengths and durations in good agreement with theory.

## Results

### The system and the Hamiltonian

Our driven TC circuit is built in a circuit-quantum electrodynamics (QED) configuration^23^, which realizes the on-chip analogue of cavity QED. Inheriting the high scalability and controllability from microwave-integrated circuits^24^,^25^ and benefiting from the significant coherence improvement of superconducting qubits over the past decade^26^,^27^, circuit–QED systems based on superconducting qubits and resonators^28^,^29^ are suitable for building large-scale quantum simulators^30^,^31^,^32^,^33^ to study fundamental many-body problems.

**Figure 1:**
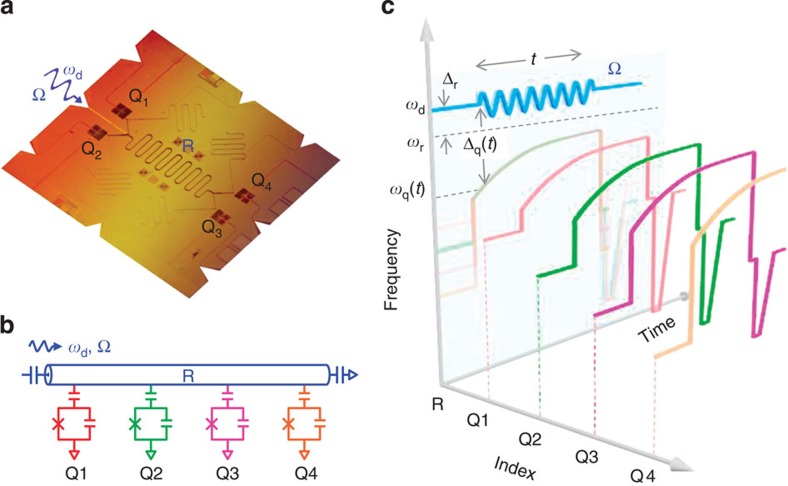
Diagrams of device and measurement sequence. (**a**) A false-colour device image highlighting the circuit elements such as the qubits (dark squares) and the half-wavelength coplanar waveguide resonator (the sinusoidal line in the middle). Four superconducting qubits *Q*_*k*_ (*k*=1, 2, 3 and 4) are individually coupled to the resonator (R). The microwave drive to the resonator is applied through the transmission line between *Q*_1_ and *Q*_2_ as indicated. (**b**) Simplified circuit schematic. (**c**) Illustration of the pulse sequence, where the *x* axis indexes the qubits and the resonator, the *y* axis represents the sequence time and the *z* axis represents the frequency (Supplementary Note 5 for designing the sequence). The four qubits, originally sitting at their idling frequencies, are simultaneously tuned to the same frequency *ω*_q_(*t*_0_) such that *λ*/*λ*_c_=0.5 (at this point all qubits and the resonator are individually in their own ground state), following which *ω*_q_(*t*) is swept for a time *t* up to *τ*, such that *λ*/*λ*_c_ increases uniformly from 0.5 to 2.5 over the full period of *τ* (see the asymptotic curves and their shades). During the ramping, a microwave drive (the blue sinusoidal line) to the resonator R with a fixed frequency *ω*_d_ and a fixed drive strength Ω is always on (Methods for determining Ω). We record the four-qubit occupation probabilities as functions of the sweep time *t*, by simultaneously tuning all four qubits to their measurement points at lower frequencies for joint qubit-state readout after sweeping *ω*_q_(*t*) (see the sharp trapezoids and their shades): in each sequence we record each qubit's state by ‘0' or ‘1' in a single-shot manner, and repeating the same sequences many times ∼10^3^–10^4^), we count the 16 probabilities *P*_0000_, *P*_0001_, *P*_0010_, ⋯ and *P*_1111_, where ‘0' and ‘1' denote, respectively, the ground and excited states of each qubit. These probabilities are used to calculate the collective spin operator 〈*J*_*z*_〉 (Methods).

Figure 1a,b presents the circuit layout, which consists of four superconducting phase qubits coupled to a common coplanar waveguide resonator^19^. The resonator frequency is fixed at *ω*_r_/2*π*≅6.2 GHz, around which the resonance frequency of each qubit (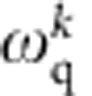 for *k*=1, 2, 3 or 4) can be individually adjusted. The resonator's energy decay rate is *κ*_1_≅0.4 MHz and its pure dephasing rate *κ*_2_ is negligible. Because the energy decay rate and the pure dephasing rate for the qubits slightly vary as functions of qubit frequency, we sample their values in a frequency range from 6 to 6.15 GHz and take the average in numerical simulation. The qubits' energy decay rates are, on average, Γ_1_≅2.0 MHz and their pure dephasing rates are, on average, Γ_2_≅4.0 MHz. Couplings between each qubit and the resonator are fixed by designing the coupling capacitors to be nearly identical and therefore we consider a homogenous coupling strength *λ*/2*π*=30 MHz in the following treatment (Supplementary Note 5 and Supplementary Fig. 1 for detailed sample parameters).

Applying an external microwave tone at *ω*_d_, we may generally describe the Hamiltonian of the system in a rotating frame as,


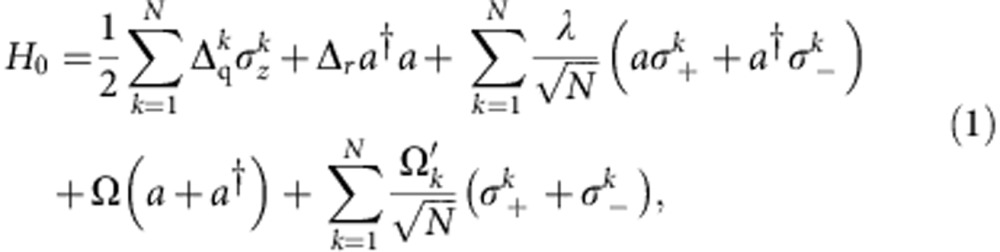


where *N*=4, 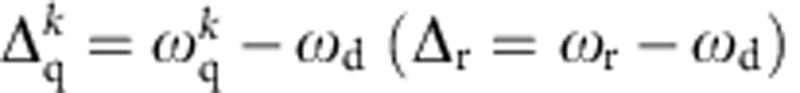 is the detuning of the qubit (resonator) resonance from the drive frequency, *a* (*a*^†^) is the lowering (raising) operator of a single mode of the resonator and 
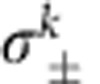
,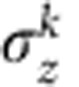 are the *k*^th^-spin Pauli operators. Ω and 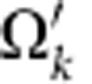 are, respectively, the driving strengths to the resonator and to the *k*^th^ qubit. To understand the nonequilibrium QPT as derived from equation (1), we assume four identical spins for simplicity, and we simultaneously steer all four qubits on the same frequency trajectory *ω*_q_(*t*) as the system evolves, yielding 
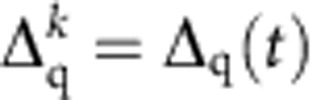
. This assumption applies to our experiment (Supplementary Fig. 5) and reduces the complexity due to parametric inhomogeneity^34^,^35^. Under another homogeneous approximation, that is, 
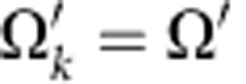
, the last two terms in equation (1), regarding the drivings on the resonator and the qubits, are unitarily equivalent^22^. Since our microwave tone in the present experiment is designed to drive the resonator, the effect due to driving the qubits via the unwanted but small microwave crosstalk can be absorbed into that of driving the resonator. As a result, equation (1) is simplified by neglecting the small terms involving 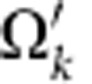 in the following treatment.

The original undriven TC model possesses, in theory, a critical coupling at 
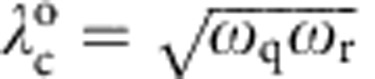
, which is impossible to reach in our circuit. In a rotating-frame variant of the driven TC Hamiltonian shown in equation (1), the qubit (resonator) resonance frequency *ω*_q_ (*ω*_r_) is replaced by the associated detuning Δ_q_ (Δ_r_), yielding a QPT whose critical point now scales with the geometric mean of the spin and field detunings, that is, 
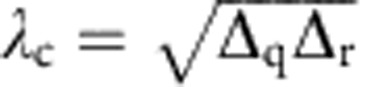
. For such an off-resonantly driven TC system, the QPT can be traversed by engineering the homogenous coupling strength *λ* to pass through *λ*_c_.

**Figure 2:**
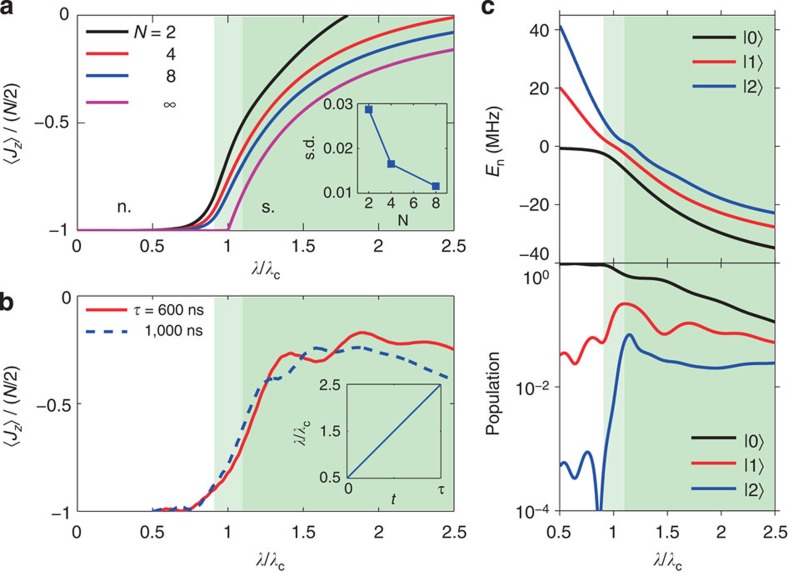
〈*J*_*z*_〉's signature behaviours across the critical point in the ground-state quantum phase transition and in the experimental dynamics, calculated with Δ_r_/2*π*=30 MHz and Ω/2*π*=4 MHz for example. The quantum critical region, illustrated by the light-green background in all panels, happens around *λ*/*λ*_c_=1 between the normal (n., the white region) and super-radiant (s., the green region) phases. (**a**) Numerical calculations of 〈*J*_*z*_〉/(*N*/2) by solving equation (1) for the ground state at different number of qubits *N* as indicated. The cusp-like behaviour at the critical point *λ*/*λ*_c_=1 occurs only in the thermodynamical limit, and the finite qubit cases (*N*=2, 4 and 8) display the drastic rise after traversing the critical point. Inset illustrates the maximal standard deviations (s.d.) of 〈*J*_*x*_〉/(*N*/2) as calculated in **a** because of random noise (or inhomogeneity) for different number *N* of qubits involved. For illustrative purpose, here we only consider the frequency uncertainty in each qubit 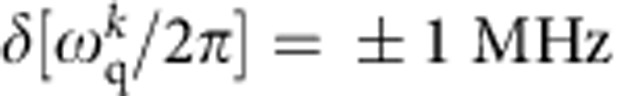, relevant to our experimental set-up. It is seen that uncertainties do not give large errors and increasing the number of qubits yields better suppression of the random noise. (**b**) Numerical calculations of 〈*J*_*z*_〉/(*N*/2) as function of *λ*/*λ*_c_ following the experimental pulse sequence in Fig. 1c at different durations as indicated. Sample decoherence is included in calculations. It is seen that 〈*J*_*z*_〉/(*N*/2) curves rise around the same point as that in **a**. Inset illustrates *λ*/*λ*_c_ as a function of the ramping time *t* during the pulse sequence. (**c**) Numerically calculated energies of the lowest three energy eigenstates (top) and population distribution (in logarithmic scale) among these three states (bottom) of the four-qubit Hamiltonian system described in equation (1) as functions of *λ*/*λ*_c_ under decoherence, with *τ*=600 ns. Higher-energy states are omitted for clarity. Starting with all qubits and the resonator in their own ground states at *λ*/*λ*_c_=0.5 (at this point the system's ground state |0〉 is at *E*_0_≈0 and takes the largest population as shown by the black line), *E*_*n*_ of the lowest few states significantly drop below 0 and the population distribution quickly evolves as *λ*/*λ*_c_ increases above 1 (in the light-green region), indicating a structural change of the eigenstates of the system crossing this critical point.

Since λ is fixed in our case, to experimentally observe the QPT, we sweep *λ*_c_ through *λ*, that is, we sweep Δ_q_ such that *λ*/*λ*_c_ increases linearly with time from 0.5 to 2.5 over a duration of *τ* (see Fig. 2b inset). The detailed pulse sequence for performing the experiment is illustrated in Fig. 1c: starting with all qubits and the resonator in their own ground states, we turn on the microwave drive at a fixed resonator-drive detuning Δ_r_ and then immediately tune all four qubits to the same frequency such that *λ*/*λ*_c_=0.5. Following this, we sweep the qubit frequency on an asymptotic trajectory (achieving a constant ramping rate for *λ*/*λ*_c_) for a time duration *τ*. Dynamics of the system during the ramping of *λ*/*λ*_c_ are measured by recording the four-qubit joint occupation probabilities as functions of the sweep time. Evidence of the QPT can be witnessed in the change of the inferred mean values of the collective spin operator, that is, 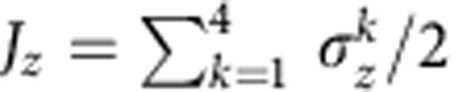, as *λ*/*λ*_c_ increases above 1.

We note that our qubit is not an exact spin-1/2 system because of its weak anharmonicity, that is, there exists a next higher energy state. The pulse sequence, shown in Fig. 1c, is designed to avoid significant state population leakage to the qubit's next higher energy state. When probing the four-qubit dynamics we specifically parametrize some relevant Hamiltonian parameters such as the drive strength Ω/2*π*, the resonator-drive detuning Δ_r_/2*π* and the total sweep duration *τ* under experimental constraints (Supplementary Note 5 for detailed discussions).

### The QPT in the driven TC model

Before presenting our experimental results, we first describe the ideal QPT as predicted by theory^22^, and relate it to our experimental reality. In particular, we try to clarify the quantum critical behaviour in the context of a few qubits coupled to a common resonator mode and discuss the connection between the few-qubit case and the case in the thermodynamic limit; we also try to clarify how the dynamical measurement via a swept *ω*_q_(*t*) (equivalent to uniformly varying *λ*/*λ*_c_ over time) correlates with a signature of the QPT. According to ref. 22, the QPT is present when the system switches from a normal phase to a super-radiant phase in the rotating frame. In this generic ground state QPT, around the QPT's critical point (*λ*/*λ*_c_=1) we may observe a sharp cusp in the scaled moments 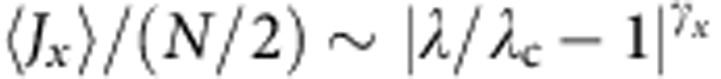 (with *γ*_*x*_=1/2) and 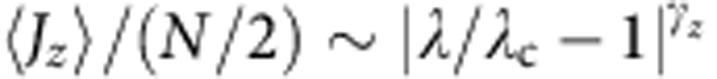 (with *γ*_*z*_=1) for *λ*/*λ*_c_⩾1, and also in the mean number of photons with 
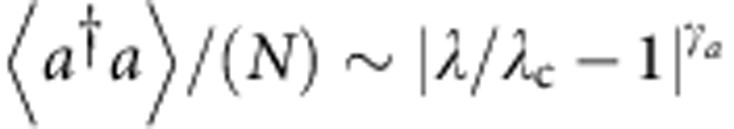
 (with *γ*_*a*_=1) for *λ*/*λ*_c_⩾1. The critical exponents *γ*_*x*,*z*,*a*_ represent the critical scaling behaviour observable in the thermodynamical limit (Supplementary Note 1). In contrast to the cusp-like behaviour in the thermodynamic limit, the QPT in the few-qubit case yields 〈*J*_*z*_〉/(*N*/2) curves that rise in a smooth but abrupt manner (Fig. 2a). Nevertheless, the critical point for the few-qubit case can still be visually identified proximal to *λ*/*λ*_c_=1. Owing to the dissipative nature of our system and the hardware limitation we focus our observation on 〈*J*_*z*_〉/(*N*/2), whose behaviour around *λ*/*λ*_c_=1 can be a sufficient evidence of the QPT (Discussion and Methods).

The QPT as evidenced in Fig. 2a, by the rise of 〈*J*_*z*_〉/(*N*/2), is a generic ground-state QPT in the rotating frame^22^. Starting from the normal ground state at *λ*/*λ*_c_<<1, to reach the super-radiant ground state at *λ*/*λ*_c_>1 we have to ramp up *λ*/*λ*_c_ very slowly, in accordance with the adiabatic condition. For an open quantum system, the adiabatic condition can be difficult to satisfy since dissipation plays a decisive role, given long enough evolution times. As such, we examine the QPT in a nonadiabatic manner: we ramp up *λ*/*λ*_c_ quickly and linearly over durations that range from a few hundred to a thousand nanoseconds (comparable to the qubit energy relaxation time 1/Γ_1_), in order to minimize the impact of dissipation on the dynamics. During the process we constantly monitor the four-qubit occupation probabilities, from which we calculate 〈*J*_*z*_〉/(*N*/2) to study its behaviour over time. As a comparison, we numerically model the time evolution of 〈*J*_*z*_〉/(*N*/2) under open system dynamics as described by equation (1) based on a master equation approach (Supplementary Note 3). As our numerical simulation suggests, excited states of the system can be populated during the evolution, and the population distribution among different eigenstates tends to stabilize after *λ*/*λ*_c_ increases above 1.5 (Fig. 2c). In particular, in the nonadiabatic process and under decoherence, 〈*J*_*z*_〉/(*N*/2) still rises up around *λ*/*λ*_c_=1, in a style (Fig. 2b) very similar to that in Fig. 2a. Therefore, the onset where 〈*J*_*z*_〉/(*N*/2) rises up abruptly from −1 should correlate well with the critical point of the generic ground-state QPT, which in itself reflects a situation where a qualitative change occurs in the properties of the system's eigenstates as a function of the Hamiltonian parameter in equation (1) (here 
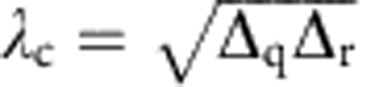
). Our experiment, although involving the system's higher energy states, should still provide strong evidence for the QPT via the observed abrupt change of 〈*J*_*z*_〉/(*N*/2) as *λ*/*λ*_c_ is tuned through unity.

### Experimental observation of the QPT

**Figure 3:**
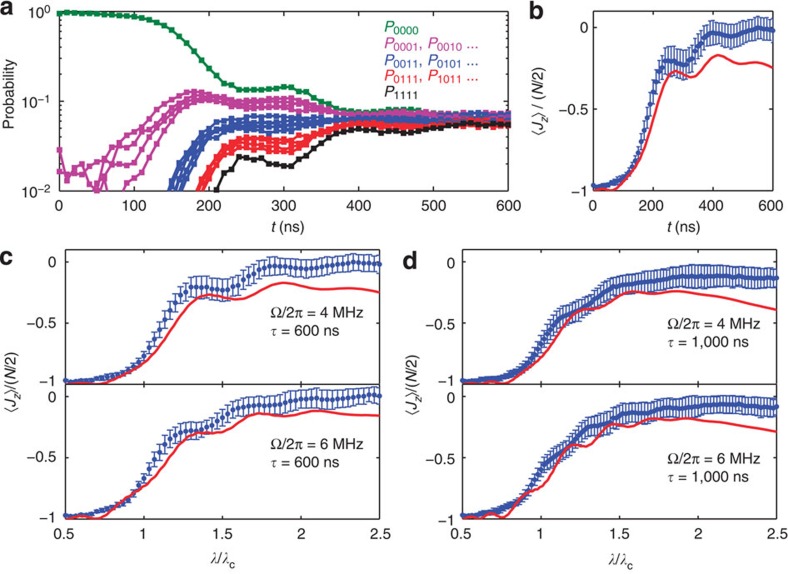
The four-qubit quantum phase transition experimental results in comparison with numerical simulation. (**a**) Four-qubit joint occupation probabilities (in logarithmic scale) as functions of the sweep time *t* for Δ_r_/2*π*=−30 MHz, Ω/2*π*=4 MHz and *τ*=600 ns (error bars, on the order of 1%, are not shown for clarity). *λ*_*c*_/2*π* varies from 60 MHz at *t*=0 ns to 12 MHz at *t*=600 ns. The 16 probabilities are grouped by their corresponding excitation quanta as marked by different colours: green for no excitation (*P*_0000_), purple for one quantum excitation (*P*_0001_, *P*_0010_, *P*_0100_, *P*_1000_), blue for two quantum excitation (*P*_0011_, *P*_0101_, ⋯, *P*_1100_), red for three (*P*_0111_, *P*_1101_, *P*_1011_, *P*_1110_) and black for four (*P*_1111_). The critical point is approached when the two-quantum-excitation curves start to gain finite probability values. Curves in the same group behave similarly, validating the identical spin assumption in the QPT theory. (**b**) 〈*J*_*z*_〉/(*N*/2) dynamics calculated from data in **a** (points with error bars). Line is a numerical simulation. Error bars are s.d. of repetitive measurements, during each measurement we add a random bias sequence to each qubit to simulate the frequency uncertainties of ±1 MHz (the uncertainty level of our calibration of the qubit frequency). Experimentally measured error bars agree with numerical calculations considering all known uncertainties in our experiments, with the majority of the errors coming from the frequency uncertainties in biasing the qubits, which accumulate over the sweep time, and the readout uncertainties of the occupation probability. (**c**,**d**) 〈*J*_*z*_〉/(*N*/2) as functions of *λ*/*λ*_c_ showing the existence of QPT (points with error bars). Lines are numerical simulations including decoherence. Error bars are obtained similarly to those in **b**. The choice of a negative Δ_r_ is to experimentally minimize the state leakage, which should not affect the dynamics and the QPT physics as calculated using a positive Δ_r_ in Fig. 2. Experimental signal of 〈*J*_*z*_〉 is slightly larger than theory prediction because of the slight state leakage, which is miscounted as 〈*J*_*z*_〉's signal in the measurements (Supplementary Figs 3 and 4).

Following the experimental sequence outlined in Fig. 1c, in Fig. 3a we show the typical dynamics measured at Δ_r_/2*π*=−30 MHz, Ω/2*π*=4 MHz and *τ*=600 ns, with the 16 four-qubit joint occupation probabilities (*P*_0000_, *P*_0001_, *P*_0010_,⋯) evolving with the sweep time *t*. The choice of a negative Δ_r_ is to minimize the state leakage caused by the microwave drive, which should not affect the dynamics and the QPT physics as calculated using a positive Δ_r_ in Fig. 2. The 16 probabilities can be grouped according to their excitation quanta, and the very close dynamics of the probabilities in the same group suggest that four qubits behave similarly, validating the identical spin assumption in the QPT theory. 〈*J*_*z*_〉/(*N*/2) can be calculated using these 16 probabilities, as processed in Fig. 3b (points with error bars). By mapping the *x* axis in time to *λ*/*λ*_c_, we display the 〈*J*_*z*_〉/(*N*/2) versus *λ*/*λ*_c_ curves, with values of Ω as listed and Δ_r_/2*π*=−30 MHz, for the cases of *τ*=600 and 1,000 ns in Fig. 3c,d, respectively (more experimental data for Δ_r_/2*π*=−20 MHz can be found in Supplementary Fig. 2). Comparing with those shown in Fig. 2a,b, it is seen that the experimental results have unambiguously caught the main feature of the off-resonantly driven QPT, that is, a signature rise of the scaled moment 〈*J*_*z*_〉 as *λ*/*λ*_c_ increases above 1, the critical point. We note that the spectral line widths for the qubits and the resonator are defined by their energy relaxation and dephasing rates (Γ_1_, Γ_2_, *κ*_1_ and *κ*_2_), all less than values of |Δ_q_| (for example, ≈2*π* × 13 MHz at *λ*/*λ*_c_=1.5 where 〈*J*_*z*_〉 rises to a high level. Note that Δ_q_<0) and |Δ_r_| (=2*π* × 30 MHz) used in measurements for data in Fig. 3. We also verify that state leakage to the next higher energy state of the qubits is reasonably small during these measurements (Supplementary Fig. 3). As such, the rise around *λ*/*λ*_c_=1 is compatible with the critical point quoted in the context of the nonequilibrium QPT, which reflects a structural change of the system's eigenstates.

Different from the ideal QPT case in the isolated system, the interplay between the external drive and decoherence irreversibly evolves the system into a nonequilibrium quasi-steady state, where the term quasi refers to the fact that 〈*J*_*z*_〉/(*N*/2) tends to approximately level off at longer sweep times *τ*. Using typical coherence parameters of our device, we also simulate the experimental conditions for the experimental data shown in Fig. 3b–d (lines). The numerical results show that the system reaches the quasi-steady state approximately after *λ*/*λ*_c_>1.5, and the situation slightly varies with Δ_r_. Nevertheless, our experimental data are in good agreement with numerical simulations taking into account decoherence with no fitted parameters.

Moreover, a faithful simulation requires a clear understanding of the operational imperfections, particularly when the underlying problem is otherwise intractable. As discussed in the Supplementary Note 5, we specifically design the pulse sequence to minimize the dominant experimental imperfections in equation (1), including using appropriate negative detunings of {Δ_r_, Δ_q_} and ramping rates of *λ*/*λ*_c_ (or equivalently the sweep durations *τ*). Nevertheless, there are other experimental subtleties that we cannot avoid, for example, slight state leakage (miscounted as 〈*J*_*z*_〉's signal in the measurements). By taking into account of the state leakage, we find better agreement between the experimental data and theory (details in the Supplementary Note 5).

## Discussion

To further understand quantum critical behaviour beyond the observed QPT, we have to return to the noiseless model. We first refer to an undriven TC model in comparison with the driven TC model (equation (1)), in the latter of which the ground-state QPT is related to a breaking of the parity symmetry^22^. The undriven TC Hamiltonian 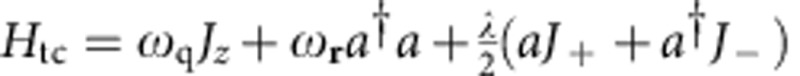 commutes with a parity operator *P*=*e*^*iπL*^, where *L*=*J*_*z*_+*a*^†^*a*+*N*/2 represents the total number of excitations of the collective system. Parity conservation ensures that 〈*J*_*x*_〉 remains zero and 〈*J*_*z*_〉 increases in a staircase manner as the spin-field coupling *λ* increases. With increasing *λ*, it is shown^22^ that level crossings occur, for example, between the ground state and the excited states, after crossing the critical point (that is, 
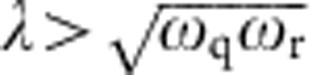
 purely for theoretical discussion only. Note that by definition 
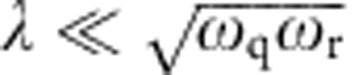
 is required in the TC model), and this results in discrete parity changes in the state of the system. However, in the driven TC model, with critical point changed from 
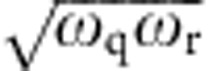
 to 
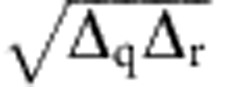
, the QPT really happens; however, parity is no longer conserved since [*H*_0_,*P*]≠0. The broken parity can be associated with avoided level crossings in the eigenspectra, and the excitation number *L* is no longer conserved. This results in a smooth rise, that is, a ‘rounded' staircase for 〈*J*_*z*_〉, after crossing the critical point, if the microwave drive is weak enough (note that in the present experiment the weak drive limit is not reached since equation (1) assumes four identical spins, which requires that the drive strength Ω has to cover, at least, the frequency uncertainties while simultaneously tuning all four qubits to follow the same frequency trajectory *ω*_q_(*t*)).

The QPT under consideration also behaves differently from the Dicke QPT^10^ as regards parity. It is a generic Dicke model, rather than a driven Dicke model, achieved in ref. 10, for which parity is conserved in the normal phase. In contrast, no parity is conserved in our driven TC system in either the normal or the super-radiant phase. Parity breaking is responsible for some scaling behaviour, and the parity symmetry is relevant to various types of quantum correlations^36^,^37^.

The driving in our case not only breaks the parity symmetry of the original TC system, but also helps circumventing the ‘no-go' theorem due to the restriction from the Thomas–Reiche–Kuhn sum rule^38^,^39^,^40^. As discussed in Supplementary Note 2, the small *A*^2^ term (with *A*^2^=*κ*(*a*+*a*^†^)^2^), which is neglected in the above treatment but whose appearance might forbid the QPT, turns to be a harmless shift in Δ_r_ in the rotating frame. Therefore, the introduction of the driving profoundly alters the resulting physics, enabling the observation of a nonequilibrium QPT.

Although our data are fully compatible with the nonequilibrium QPT picture as predicted by the off-resonant driven TC theory, it is worth noting that currently we cannot exclude the possibility of a semiclassical interpretation of our experiment. In contrast to our experimental condition that involves only finite quantum elements and finite excitation levels in each element, semiclassical treatments employ continuous variables that would work better in the thermodynamic limit. Unfortunately, the relevant semiclassical treatments that we are aware of only deal with specific conditions and are not suitable for interpreting our off-resonant driven TC experiment (Supplementary Note 4). Therefore, whether a semiclassical alternative is possible to explain the data remains an open question. Along this route, the tomography measurement of the QPT dynamics, although technically challenging, could allow the exploration of any possible quantum correlations encoded in the QPT, which would be useful to answer the open question as regards a semiclassical alternative in future experiments.

In addition, following on from this work we expect demonstrations of a staircase behaviour in 〈*J*_*z*_〉 and a cusp-like behaviour in 〈*J*_*x*_〉 around the critical point, both hallmarks of the generic ground-state QPT, in future experiments using larger numbers of closely identical qubits with improved coherence and more sophisticated control. By further suppressing decoherence we may enable the demonstration of the ground-state QPT in a configuration similar to the present device strictly following the proposed implementation in ref. 22. With recent progress in superconducting quantum information technology and the promising outlook to develop intermediate-scale complex quantum circuits, we believe that further exploration of many-body physics in a nonequilibrium condition by building a solid-state quantum simulator with only weak spin-field couplings can be expected in the near future. This will help improve our understanding of the interplay between nonequilibrium and quantum correlations as well as the role of parity symmetry in many-body systems.

## Methods

### Tuning Ω

The microwave drive strength Ω in equation (1) depends on both the microwave drive amplitude *A* and the coupling capacitance for feeding energy into the resonator, with the latter being set once the device and the measurement set-up are fixed. *A* is what we usually quote using the room-temperature electronics. More importantly, the Ω–*A* relation could weakly depend on the off-resonance magnitude of Δ_r_ because of the frequency dependence of the transmission coefficient of the microwave cables at cryogenic temperatures, and the interference by various box modes and spurious two-level defect modes. To find out the exact Ω used in our experiment, we start with determining the on-resonance Ω by calibrating the relation between Ω and *A*. We resonantly drive the resonator using a single-tone microwave pulse with an amplitude *A* for a period of *t* (typically 50 ns), after which we bring a qubit, originally in its ground state, to resonantly interact with the resonator for detecting the resonator state^24^. The microwave drive generates a coherent state in the resonator and the subsequent qubit–resonator interaction results in multitone vacuum Rabi oscillations whose frequencies depend on the resonator populations. We record the time evolution of the qubit probabilities in the excited state for the first 300 ns, from which the energy-level population probabilities of the resonator (*P*_*n*_ for *n*=0, 1, 2, ⋯) are inferred. The resonator energy-level populations satisfy the Poisson distribution and we quote the displacement *α*, which is the square root of the average photon number in the resonator, by *α*=(∑_*n*_*nP*_*n*_)^1/2^. For a fixed experimental set-up and a fixed *t*, the ratio *γ*=|*α*|/*A* is a constant, and can be experimentally determined by sampling a group of *A* and *α* values. The drive strength is thus Ω=*γA*/*t*.

To calibrate the off-resonance Ω, we carry out the 2-qubit experiment with a similar set-up as discussed in Fig. 1, using the on-resonance Ω value (=2*π* × 4 MHz) as an initial trial. The measured results are then compared with numerical simulation, which verifies that Ω values calibrated on resonance are also applicable at small detuning values of Δ_r_ used in the four-qubit experiment (Supplementary Fig. 6 for more detail).

### Qubit readout and the correction

The qubit readout is performed using an integrated superconducting quantum interference device (SQUID), which can tell the flux difference between the qubit's ground and excited states. The readout details can be found in ref. 41. We simultaneously read out all the states of the four qubits (one SQUID for each qubit), therefore, obtaining the 16 probabilities *P*_0000_, *P*_0001_, *P*_0010_,..., *P*_1111_. These values are corrected before being further processed. The readout fidelities for |*g*〉 (*F*_*k*,*g*_) and |*e*〉 (*F*_*k*,*e*_) for qubit *Q*_*k*_ are obtained using the single-qubit measurement. The correction matrix for *Q*_*k*_ is the inverse of


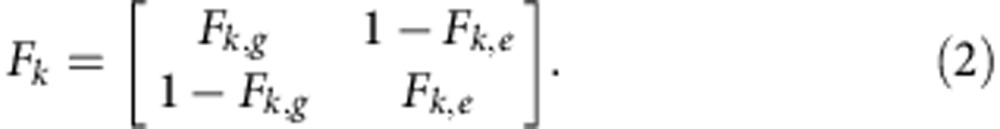


We correct all 16 values using the inverse of the tensor–product matrix 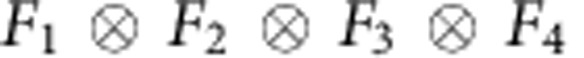. The correction matrix may be slightly off because of the small flux crosstalk when simultaneously reading out all four qubits, which is a possible reason for that the experimental 〈*J*_*z*_〉/(*N*/2) value does not start from −1.0 at *λ*/*λ*_c_=0.5 in Fig. 3.

### Expectation values of the spin operator *J*_*z*_

After correcting the 16 qubit-state probabilities, we calculate the scaled 〈*J*_*z*_〉 using


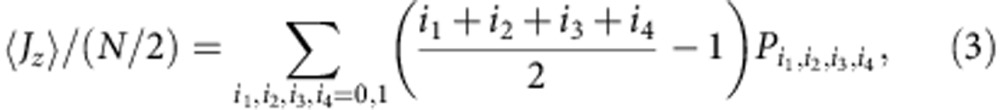


where *i*_*k*_=0, 1 represents the ground and excited states of qubit *Q*_*k*_, respectively, and the summation runs over all four-qubit eigenstates corresponding to the 16 probabilities.

To calculate 〈*J*_*x*_〉/(*N*/2), the four-qubit state tomography must be performed, which requires *π*/2 rotations on all four qubits (in addition to the microwave tone on the resonator) before readout. We are unable to measure 〈*J*_*x*_〉/(*N*/2) mainly because of our limited hardware resource. In addition, the involved dynamical phase when performing the tomography could cause extra complexity in calculating 〈*J*_*x*_〉/(*N*/2). Since *J*_*z*_ is not affected by the dynamical phase and its rise traversing *λ*/*λ*_c_=1 can be sufficient proof of the QPT, we choose to only measure 〈*J*_*z*_〉/(*N*/2) in the experiment.

## Additional information

**How to cite this article:** Feng, M. *et al*. Exploring the quantum critical behaviour in a driven Tavis–Cummings circuit. *Nat. Commun.* 6:7111 doi: 10.1038/ncomms8111 (2015).

## Supplementary Material

Supplementary Information Supplementary Figures 1-6, Supplementary Notes 1-5 and Supplementary References
