# Supplementary material for: Exploring the quantum critical behaviour in a driven Tavis–Cummings circuit
Source: Nat Commun. 2015 May 14;6:7111. doi: 10.1038/ncomms8111 (PMC4479029; doi:10.1038/ncomms8111)
Supplement: Supplementary Information — Supplementary Figures 1-6, Supplementary Notes 1-5 and Supplementary References [file ncomms8111-s1.pdf]

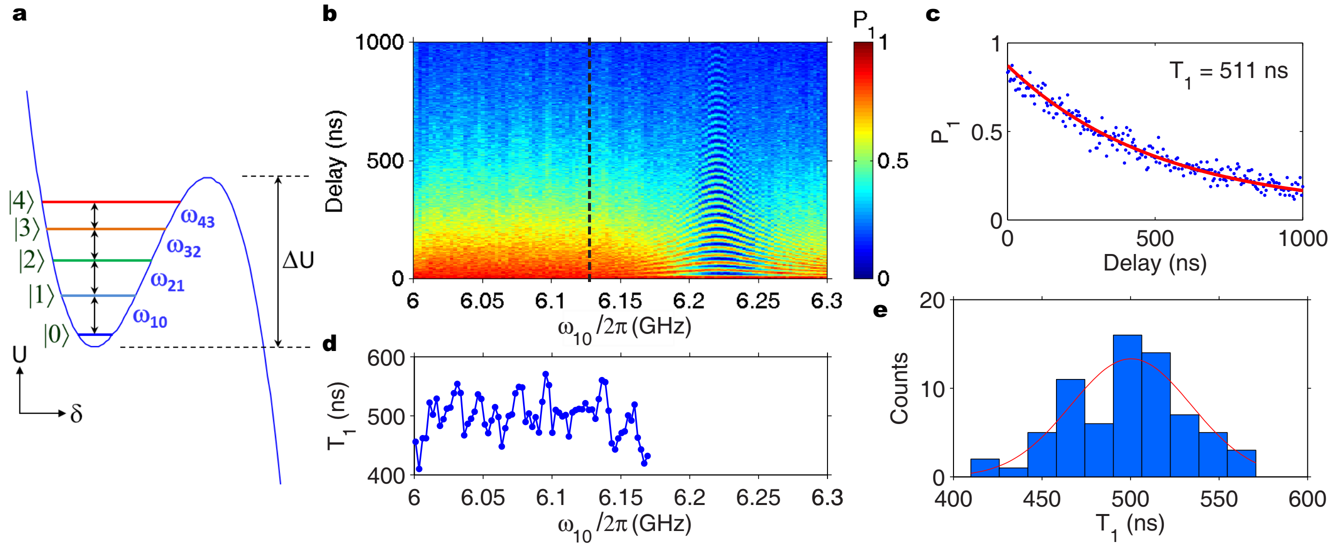

Supplementary Figure 1: The cubic potential of the phase qubit and its energy relaxation T_1. (**a**) Illustration of the cubic potential as a function of the junction phase δ in a Josephson-junction device with, e.g., five energy states. The two-level phase qubit is encoded using the two lowest energy states $|0\rangle$ and $|1\rangle$. Transition frequencies $\omega_{n,n-1}/2\pi$ are separated from each other by about 100 MHz. (**b**) The swap spectroscopy measurement showing the qubit decay rate at different frequencies: after exciting the qubit to $|1\rangle$, the qubit is biased to a fixed frequency (the x-axis) and its $|1\rangle$-state probability P_1 (color scale) is measured after a delay time (the y-axis). The Lorentzian-shaped chevron indicates the qubit-resonator interaction and its center position is the resonator frequency. (**c**) A typical T_1 decay curve (points) obtained along the dashed line in (**a**) at $\omega_q/2\pi \equiv \omega_{10}/2\pi = 6.129$ GHz. Line is a fit with $T_1 = 511$ ns. (**d**) Fitted T_1 values as a function of the qubit frequency as obtained from data in (**a**). T_1 values nearby the resonator frequency are not shown since a simple decay fit is not accurate in this region. (**e**) A histogram analysis of the T_1 values in (**c**). Line is a Gaussian fit with a mean value of 500 ns and a standard deviation of 30 ns.

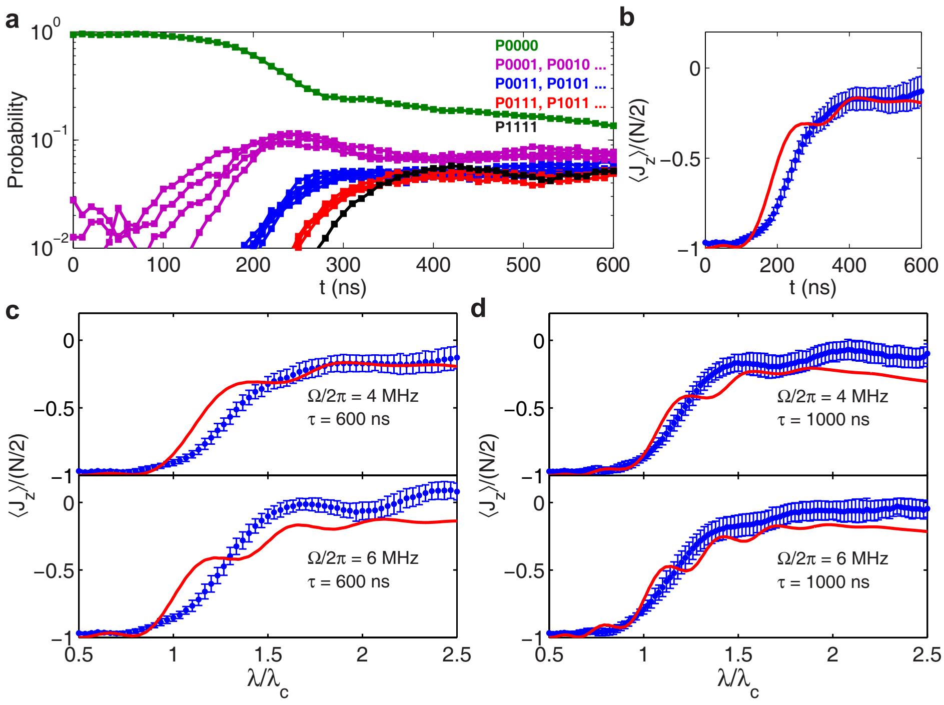

Supplementary Figure 2: The four-qubit ($N = 4$) QPT experimental results in comparison with numerical simulation at $\Delta_r/2\pi = -20$ MHz. (**a**) Four-qubit joint occupation probabilities (in logarithmic scale) as functions of the sweep time t for $\Omega/2\pi = 4$ MHz and $\tau = 600$ ns (error bars, on the order of 1%, are not shown for clarity). The sixteen probabilities are grouped by their corresponding excitation quanta as marked by different colors. (**b**) $\langle J_z \rangle/(N/2)$ dynamics calculated from data in (**a**) (points with error bars). Line is a numerical simulation. Error bars are standard deviations of repetitive measurements, obtained similarly to those in Figure3 of the main text. (**c, d**) $\langle J_z \rangle/(N/2)$ as functions of λ/λ_c showing the existence of QPT (points with error bars). Lines are numerical simulations including decoherence. Error bars are obtained similarly to those in **b**. Slightly larger difference between the experimental data and numerical simulation as compared with the $\Delta_r/2\pi = -30$ MHz case (Figure 3 of the main text) is likely due to more serious state leakage and less accuracy in controlling (in particular for $\tau = 600$ ns) the qubit trajectory $\omega_q(t)$ at $\Delta_r/2\pi = -20$ MHz.

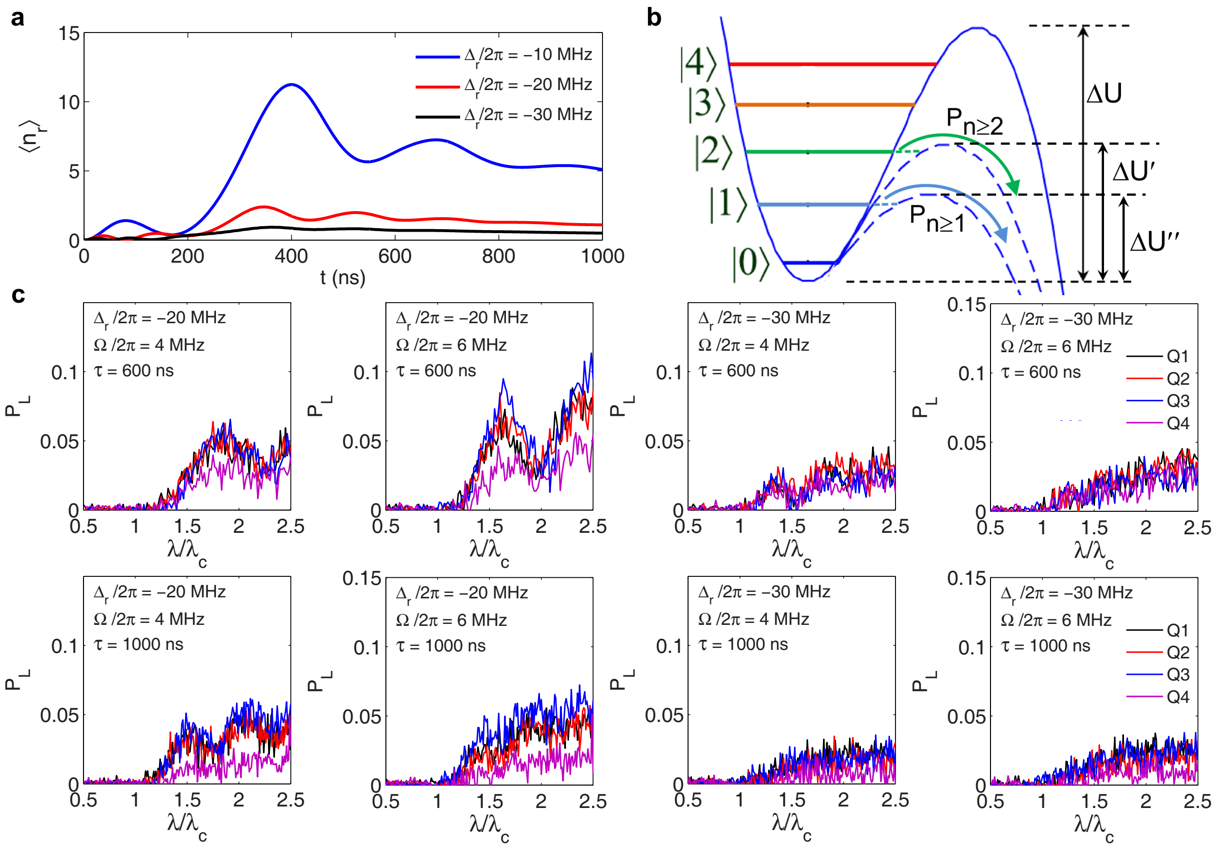

Supplementary Figure 3: Characterizing the state leakage probability P_{L}, i.e., sum of occupation probabilities in the $|2\rangle$ and higher excited states of each qubit circuit. (**a**) The average photon number $\langle n_{\mathrm{r}}\rangle$ in the resonator accumulated during pulse sequences of $\tau = 1000$ ns and $\Omega/2\pi = 4$ MHz as revealed by the master equation simulation. It is seen that $\langle n_{\mathrm{r}}\rangle$ at $\Delta_{\mathrm{r}}/2\pi = -10$ MHz increases quickly with the sweep time t, which may lead to serious state leakage via the qubit-resonator photon swap process. As such $\Delta_{\mathrm{r}}/2\pi = -10$ MHz should be avoided experimentally. (**b**) Illustration showing the procedure of obtaining P_{L} by properly lowering the potential barrier to $\Delta U'$ ($\Delta U''$) and measuring the corresponding tunneled probability $P_{n\geq 2}$ ($P_{n\geq 1}$). $P_{\mathrm{L}} \approx P_{n\geq 2} - 0.05 \cdot (P_{n\geq 1} - P_{n\geq 2})$, where the factor 0.05 corresponds to the stray tunneled probability when the qubit is in $|1\rangle$ and the potential barrier is lowered to $\Delta U'$. (**c**) Experimentally obtained P_{L} for each qubit circuit (legend on the far right) as functions of $\lambda/\lambda_{\mathrm{c}}$ for data shown in Figure 3 of the main text and in Supplementary Figure 2. Parameters are as labeled. The small values of P_{L} indicate that Supplementary Equation 3 involving two-level qubits is a qualified model to describe the experimental situation.

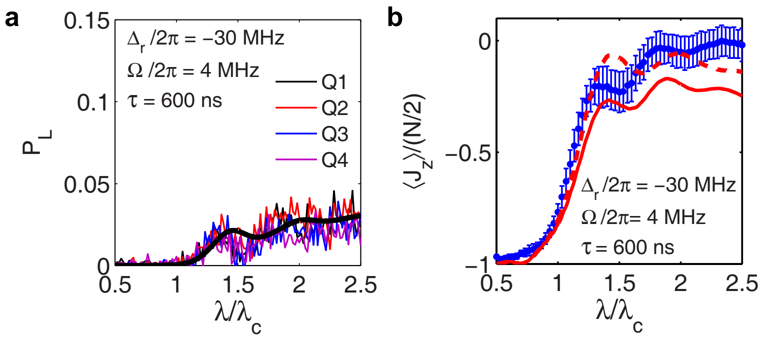

Supplementary Figure 4: Numerical simulation of $\langle J_z \rangle$ considering three energy levels in the qubit circuit (a qutrit). (**a**) Numerical simulation of P_L (the thick black line), which is P_2 in the qutrit model, in comparison with the experimentally obtained data (thin lines). In numerical simulation the four qubits are assumed to be identical. (**b**) Numerical simulation of $\langle J_z \rangle/(N/2)$ using the qutrit model (the dashed line) as compared with the experimental data (points with error bars) and the simulated $\langle J_z \rangle/(N/2)$ curve using the qubit model (the solid line).

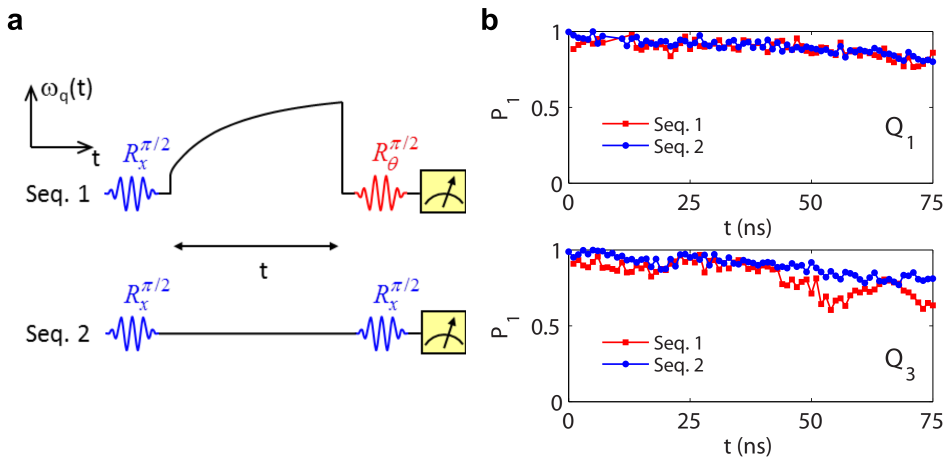

Supplementary Figure 5: Ramsey-interference-like measurement to verify the accuracy in tuning $\omega_{\rm q}(t)$. (**a**) Pulse sequence for the Ramsey-interference-like measurement with the phase of the second $\pi/2$ pulse given in Supplementary Equation 10 (Seq. 1), which is equivalent to the Ramsey interference measurement using two identical rotation pulses (Seq. 2) if the steering of $\omega_{\rm q}(t)$ is as expected. (**b**) P_1 versus t using the two pulse sequences in (**a**) for exemplary qubits as indicated (data for Q_2 and Q_4 are similar to Q_1's). Using Seq. 1, $\omega_{\rm q}(t)/2\pi$ of each qubit is tuned from 5.87 GHz to 6.14 GHz in 75 ns. Resemblance of the two curves for the same qubit indicates that we achieve a fairly accurate dynamical control of the qubit frequency over a short period. The dip around 55 ns for Q_3 using Seq. 1 is due to the interference by a TLS at 6.06 GHz.

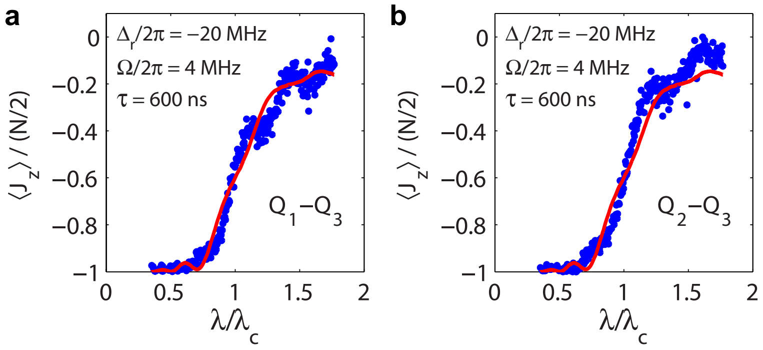

Supplementary Figure 6: Two-qubit ($N = 2$) $\langle J_z \rangle$ behaviour in comparison with numerical simulation. The two-qubit $\langle J_z \rangle/(N/2)$ versus λ/λ_c data (points) for Q_1-Q_2 (**a**) and Q_1-Q_3 (**b**). Lines are numerical simulation results. Value of Ω is calibrated via the on-resonant interaction as described in Methods of the main text.

Supplementary Note 1: Solution to the driven TC model

Unitary transformation into an interacting representation

The four-qubit system in our experiment can be described by a driven TC model in units of $\hbar = 1$,

$$H_1 = \sum_{j=1}^{4} \omega_{\rm q}^j \sigma_z^j/2 + \omega_{\rm r} a^\dagger a + \sum_{j=1}^{4} \frac{\lambda_j}{2}(a\sigma_+^j + a^\dagger \sigma_-^j) + \Omega(ae^{i\omega_{\rm d}t} + a^\dagger e^{-i\omega_{\rm d}t}), \tag{1}$$

where $\omega_{\rm q}^j$ ($\omega_{\rm r}$) is the resonance frequency of the j-th spin (the single mode of the resonator), the latter with raising (lowering) operators $a^\dagger$ (a), $\sigma_\pm^j, \sigma_z^j$ are the $j^{\rm th}$ spin Pauli operators, λ_j is the coupling strength between the j-th spin and the resonator mode, Ω ($\omega_{\rm d}$) is the strength (frequency) of the driving applied to the resonator mode. In accordance with the experimental setup, we have neglected the small term regarding the drive on the qubits, which can also be absorbed into the term of Ω under the homogeneous assumption due to the fact that the driving effects on the resonator and the qubits are unitarily equivalent [1]. In the rotating frame with respect to the driving field, the Hamiltonian is rewritten as

$$H_2 = \sum_{j=1}^{4} \Delta_{\rm q}^j \sigma_z^j/2 + \Delta_{\rm r}\, a^\dagger a + \sum_{j=1}^{4} \frac{\lambda_j}{2}\,(a\sigma_+^j + a^\dagger \sigma_-^j) + \Omega\,(a + a^\dagger), \tag{2}$$

with detunings $\Delta_{\rm q}^j = \omega_{\rm q}^j - \omega_{\rm d}$ and $\Delta_{\rm r} = \omega_{\rm r} - \omega_{\rm d}$. Considering the homogeneous case, i.e., identical qubits and couplings, we have

$$H_3 = \Delta_{\rm q}\, J_z + \Delta_{\rm r}\, a^\dagger a + \frac{\lambda}{2}\,(aJ_+ + a^\dagger J_-) + \Omega\,(a + a^\dagger), \tag{3}$$

with $\Delta_{\rm q} = \Delta_{\rm q}^j$, $\lambda = \lambda_j$ and the collective spin operators $J_z = \sum_{k=1}^4 \sigma_z^k/2$, $J_\pm = \sum_{k=1}^4 \sigma_\pm^k$. The eigen-solution of Supplementary Equation 3 can be obtained in a displaced frame by displaced Fock states [1]. In the isolated system, to see quantum phase transition (QPT), we only need to focus on the properties of the ground state in the variation of the coupling λ with respect to the critical point $\lambda_{\rm c} = \sqrt{\Delta_{\rm q}\Delta_{\rm r}}$.

In the thermodynamical limit

To understand the finite-spin QPT we have to approach the thermodynamical limit (i.e., infinite number of spins), which is fortunately solvable analytically (see Supplementary Material in [1]). Using scaled ground-state values of $\langle J_z\rangle/(N/2) = 2\beta/N - 1$ with $\beta = 0$ for $\lambda < \lambda_{\rm c}$ and $\beta = (N/2)(1 - \Delta_{\rm q}\Delta_{\rm r}/\lambda^2)$ for $\lambda \geq \lambda_{\rm c}$, we find for the latter the cusp-like behaviour around the critical point

$$\langle J_z\rangle/(N/2) = -\frac{\lambda_c^2}{\lambda^2} = \frac{2\lambda_{\rm c}}{\lambda}(\frac{\lambda}{\lambda_{\rm c}} - 1) - \frac{\lambda_c^2}{\lambda^2}(\frac{\lambda}{\lambda_{\rm c}} - 1)^2 - 1 \sim |\lambda/\lambda_{\rm c} - 1|^{\gamma_z}, \tag{4}$$

with the exponent $\gamma_z = 1$. Similarly, we have $\langle J_x\rangle/(N/2) \sim |\lambda/\lambda_{\rm c} - 1|^{\gamma_x}$ with $\gamma_x = 1/2$ and the scaled mean number of photons $\langle a^\dagger a\rangle/N \sim |\lambda/\lambda_{\rm c} - 1|^{\gamma_a}$ with $\gamma_a = 1$.

We have to mention that the above results in the thermodynamical limit are independent of the driving strength Ω, due to the fact that $\lim_{N\to\infty}(\Omega^2/N\lambda^2) \to 0$. This implies that, before reaching the critical point, the rise of the $\langle J_z\rangle$ curve due to the excitation by the driving Ω happens only in the finite-spin cases. It represents the polariton state in the normal phase, rather than in the superradiant phase. The polariton number decreases with N for $\lambda < \lambda_{\rm c}$, and is identically zero in the thermodynamic limit. Therefore, the QPT in the finite-spin case is identified only after traversing the critical point $\lambda_{\rm c}$.

Supplementary Note 2: Discussion about the involvement of A^2 term

With respect to a real light-matter interaction we have omitted an A^2 term (with $A^2 = \kappa(a + a^\dagger)^2$) in our driven TC Hamiltonian H_1. Actually, although κ is much smaller than other characteristic frequencies, it has been generally considered that A^2 term can forbid the QPT in the Dicke model due to the restriction from the Thomas-Reiche-Kuhn

(TRK) sum rule, which is called a 'no-go' theorem [2–4]. We have also noticed some recent arguments in this topic [5]. The complete Hamiltonian should be

$$H_1' = \sum_{j=1}^{4} \omega_{\mathrm{q}}^{j} \sigma_z^{j}/2 + \omega_{\mathrm{r}} a^\dagger a + \sum_{j=1}^{4} \frac{\lambda_j}{2}(a\sigma_+^{j} + a^\dagger \sigma_-^{j}) + \Omega(a e^{i\omega_{\mathrm{d}} t} + a^\dagger e^{-i\omega_{\mathrm{d}} t}) + \kappa(a + a^\dagger)^2, \tag{5}$$

where the last term is the A^2 term.

In our case, this no-go theorem is circumvented in the non-equilibrium situation. In the frame rotating with ω_{d}, Supplementary Equation 5 can be rewritten as

$$H_\Delta = \Delta_{\mathrm{q}} J_z + \Delta_{\mathrm{r}} a^\dagger a + \frac{\lambda}{2}(a J_+ + a^\dagger J_-) + \Omega(a + a^\dagger) + \kappa(2a^\dagger a + 1), \tag{6}$$

where we have assumed identical qubits and identical couplings for each qubit to the resonator, as done above in Supplementary Equation 3. This treatment of homogeneity does not change the physical essence of the problem. From Supplementary Equation 6, we know that the extra small quadratic term in Supplementary Equation 5 can be reduced to a small shift in Δ_{r} in Supplementary Equation 6, following the rotating-wave approximation. This implies that a QPT is allowed under the drive in the non-equilibrium frame as the TRK sum rule is no longer violated. Our experimental observation of the QPT in $\langle J_z \rangle$ also confirms this conclusion.

Supplementary Note 3: Numerical simulation using master equation

We now consider the influence due to decoherence, which results from the decay/dephasing effects of the phase qubits and the superconducting resonator. In our case, the time evolution of the system can be numerically simulated by the Markovian master equation in which all the decoherence effects are phenomenologically described by the following Lindblad form

$$\begin{aligned} \dot{\rho} \ &= \ -i[H_3, \rho] + \frac{\kappa_1}{2}(2a\rho a^\dagger - a^\dagger a \rho - \rho a^\dagger a) + \frac{\kappa_2}{2}(2a^\dagger a \rho a^\dagger a - a^\dagger a a^\dagger a \rho - \rho a^\dagger a a^\dagger a) \\ &\quad + \sum_{j=1}^{4} \frac{\Gamma_1^j}{2}(2\sigma_- \rho \sigma_+ - \sigma_+ \sigma_- \rho - \rho \sigma_+ \sigma_-) + \sum_{j=1}^{4} \frac{\Gamma_2^j}{2}(\sigma_z \rho \sigma_z - \rho), \end{aligned} \tag{7}$$

where $\kappa_1(\kappa_2)$ is the decay (pure dephasing) rate of the resonator, and $\Gamma_1^j(\Gamma_2^j)$ is the spontaneous emission (pure dephasing) rate of the j-th phase qubit. In the numerical simulation we assume identical decay for each qubit, with the energy relaxation time $T_1^{\mathrm{q}} \approx 500$ ns and the Ramsey dephasing time $T_2^{\mathrm{q}} \approx 200$ ns (see Supplementary Note 5 for choosing these parameters in numerical simulations), such that $\Gamma_1^j = 1/T_1^{\mathrm{q}} = 2$ MHz and $\Gamma_2^j = 1/T_2^{\mathrm{q}} - 1/(2T_1^{\mathrm{q}}) = 4$ MHz. For the resonator, we have $\kappa_1 = 0.4$ MHz and $\kappa_2 \to 0$ MHz. Considering that the resonator is initially prepared in the vacuum state and the phase qubits are in ground states, we numerically solve Supplementary Equation 7 by the Quantum-Optics Toolbox, where $\lambda/\lambda_{\mathrm{c}}$ uniformly varies from 0.5 to 2.5 using pulse durations of 600 ns and 1000 ns, respectively, and we consider the drives $\Omega/2\pi = 4$ and 6 MHz.

Supplementary Note 4: Quantum vs semi-classical treatments

The QPT in the driven TC model was investigated previously using a semi-classical approach [6], and we have also noted some recently developed approaches solving the QPT semi-classically in the light-matter interaction [7–9]. Since noise is definitely involved in our experimental observation one may question whether one can describe the experimental data semi-classically.

We argue that there has been no semi-classical solution so far to the off-resonant driven TC model under our consideration. The above mentioned semi-classical solutions are restricted by some conditions and do not apply to our model. For example, only the resonant variant of the driven TC model can be solved and understood [6], and only the generic (non-driven) TC model can be treated semi-classically [7–9] since the parity symmetry is necessary in the solutions. In contrast, for our model with detunings and under the condition of parity breaking, it is not clear whether a semiclassical treatment can be found easily to be an appropriate description.

Nevertheless, our quantum treatment has well explained the experimental data with no fitted parameters, as presented in the main text. In contrast, the semi-classical treatment employing continuous variables which works well in the thermodynamic limit may not be applied to our case with only four qubits.

Supplementary Note 5: Experimental details

Sample parameters

Our superconducting circuit consists of four phase qubits interconnected by a central resonator. The phase qubit is encoded using a Josephson-junction device, whose cubic potential as a function of the junction phase δ is illustrated in the Supplementary Figure 1a (more about the phase qubit circuit can be found in [10]). The barrier height ΔU can be tuned to change the number of energy levels in the well. The cubic anharmonicity ensures that all transition frequencies $\omega_{n,n-1} = (E_n - E_{n-1})/\hbar$ are distinct, and is the key for qubit operation, allowing microwaves at frequency $\omega_\mathrm{q} \equiv \omega_{10} = (E_1 - E_0)/\hbar$ to drive transitions between $|0\rangle$ and $|1\rangle$ while minimizing leakage to $|2\rangle$ and higher excited states. In each qubit of our device, transition frequencies $\omega_{n,n-1}/2\pi$ are separated from each other by about 100 MHz.

Different from most previous experiments for gate demonstrations and state generations where a single operation point in frequency for each qubit is enough, here we gradually sweep the qubit frequency $\omega_\mathrm{q}(t)/2\pi$ over a span of more than 100 MHz for the case of $\Delta_\mathrm{r}/2\pi = -30$ MHz, such that the coherence performance over the entire frequency range matters. For example, Supplementary Figure 1b shows the two-dimensional (2D) swap spectroscopy measurement of Q_1 near the resonator frequency, from which we can fit the qubit T_1 (as in Supplementary Figure 1c) as a function of the bias frequency over a range relevant to the experiment. It is seen that T_1 fluctuates over frequency (Supplementary Figure 1d. T_1 can be significantly reduced, where a spurious two-level state (TLS) is present), and a histogram analysis suggests that the average qubit T_1 in this frequency range is 500 ns, with a standard deviation of 30 ns (Supplementary Figure 1e). Note that due to the strong qubit-resonator interaction at very small Δ_q, T_1 measured close to the resonator, i.e., nearby the chevron in Supplementary Figure 1b, may not be very accurate. Meanwhile, *numerical simulation of the $\langle J_z\rangle$ versus $\lambda/\lambda_\mathrm{c}$ curves based on varying T_1 values for each qubit reveal results very similar to those using a single averaged value for T_1*. Therefore we quote a single value of $T_1 = 500$ ns for simplified theoretical analysis throughout the work.

For the same reason, we quote an average Ramsey $T_2 = 200$ ns for all qubits in the analysis. The dephasing process in the phase qubit is non-Markovian due to the $1/f$ nature of flux noise. As our pulse sequence is typically a few hundred nanoseconds, a Ramsey T_2 of 200 ns should be appropriate: *numerical simulation shows that small changes of T_2 would not significantly affect the rise-up magnitude and manner of the $\langle J_z\rangle$ curves across the critical point.*

$\langle J_z\rangle$ dynamics at $\Delta_\mathrm{r}/2\pi = -20$ MHz

Similar to the $\Delta_\mathrm{r}/2\pi = -30$ MHz data shown in Figure 3 of the main text, here we show those measured at $\Delta_\mathrm{r}/2\pi = -20$ MHz in Supplementary Figure 2. For example, dynamics of the sixteen probabilities at $\Omega/2\pi = 4$ MHz and $\tau = 600$ ns (Supplementary Figure 2a) are well categorized by their excitation quanta, consistent with data measured at $\Delta_\mathrm{r}/2\pi = -30$ MHz. In addition, it is observed that the $\langle J_z\rangle/(N/2)$ versus $\lambda/\lambda_\mathrm{c}$ curves at $\Delta_\mathrm{r}/2\pi = -20$ MHz (Supplementary Figures 2c and 2d) have also caught the key feature of the off-resonantly driven QPT, i.e., a signature rise of the scaled moment $\langle J_z\rangle$ as $\lambda/\lambda_\mathrm{c}$ increases above 1.

However, difference between the experiment data and numerical simulation at $\Delta_\mathrm{r}/2\pi = -20$ MHz is more apparent than that at $\Delta_\mathrm{r}/2\pi = -30$ MHz. On one hand, it is related to the fact that state leakage to higher excited states of the phase qubit is more serious at $\Delta_\mathrm{r}/2\pi = -20$ MHz as will be discussed next; on the other hand, for $\Delta_\mathrm{r}/2\pi = -20$ MHz, Δ_q has to sweep through a wider range in frequency to achieve the desired trajectory, more likely leading to errors in calculating $\lambda/\lambda_\mathrm{c}$ (in particular for shorter τ values).

Designing the pulse sequence

The pulse sequence in Figure 1c of the main text is specifically designed considering various experimental limitations. Here we discuss a few important factors other than those already discussed in the main text.

Minimizing state leakage: the driven Tavis-Cummings theory (Equation 1 of the main text) assumes spin-1/2 particles (two-level qubits) interacting with the resonator field. Our phase qubit, though intentionally operated as a two-level system by restricting it to the two lowest energy eigenstates, contains multiple energy eigenstates in its cubic potential as illustrated in Supplementary Figure 1a. Leakage to the $|2\rangle$ and higher excited states is possible during

the pulse sequence due to the phase qubit's weak anharmonicity. It is thus important to minimize state leakage for validating the spin-1/2 assumption of the phase qubit.

Since transition frequencies $\omega_{n,n-1}$ to the $|2\rangle$ and higher excited states of the qubits are smaller than $\omega_{\rm q}$, we carry out the experiment by negative values of $\Delta_{\rm q}$ and $\Delta_{\rm r}$, implying $\omega_{\rm d} > \{\omega_{\rm q}, \omega_{\rm r}\}$, to avoid chances of accidentally driving these transitions. Due to symmetry, both positive and negative values of $\Delta_{\rm q,r}$ lead to identical solutions of Supplementary Equation 3. So we may compare the experimental data (using negative detunings) with the ideal case (employing positive detunings) in Figure 2 of the main text.

State leakage can also be due to the qubit-resonator $n_{\rm r}$-photon Rabi swap, which happens in the subspace of $|1, n_{\rm r}+1\rangle$ and $|2, n_{\rm r}\rangle$ (here the first index refers to energy levels in the phase qubit and the second one is for the resonator). Coupling between the qubit's $|1\rangle$-$|2\rangle$ and the resonator's $|n_{\rm r}\rangle$-$|n_{\rm r}+1\rangle$ is approximately $\sqrt{2(n_{\rm r}+1)}\lambda$ as determined in previous experiment [11], and the photon swap probability is $\approx 2(n_{\rm r}+1)\lambda^2/\left[2(n_{\rm r}+1)\lambda^2 + (\omega_{21}-\omega_{\rm r})^2\right]$. Therefore state leakage can be significant if the average photon number $\langle n_{\rm r}\rangle$ in the resonator is large and meanwhile the qubits and the resonator are close in frequency (the same condition as $\Delta_{\rm q}$ being tuned small for $\lambda/\lambda_{\rm c}$ approaching 2.5).

The resonator's photon population can be high under either a strong drive or a weak but continuous (long-time) drive using experimental $\Delta_{\rm r}$ values. In order to minimize state leakage, we choose to keep the drive low and vary $\Delta_{\rm q}$ with time, such that the system is subject to a weak drive at small detunings of $\Delta_{\rm q}$ only for a limited amount of time. As numerically verified in Figure 2b of the main text, linearly sweeping $\lambda/\lambda_{\rm c}$ over time offers a dynamical manner to probe the QPT, without exposing the system to external microwave drive at very small detunings of $\Delta_{\rm q}$ for a long time.

Given the linear sweep of $\lambda/\lambda_{\rm c}$, we have to carefully choose values of $\Delta_{\rm r}$ to further minimize state leakage by keeping the resonator's photon population low. For example, Supplementary Figure 3a shows the numerically-simulated average photon number $\langle n_{\rm r}\rangle$ in the resonator as functions of the sweeping time t using the pulse sequence parameters of $\tau = 1000$ ns and $\Omega/2\pi = 4$ MHz. It is seen that $\langle n_{\rm r}\rangle$ quickly accumulates with the sweep time t at $\Delta_{\rm r}/2\pi = -10$ MHz, which could incur strong qubit-resonator photon swap and should be avoided experimentally. Therefore we perform the experiment with $\Delta_{\rm r}/2\pi = -20$ MHz and -30 MHz.

With the negative $\Delta_{\rm q,r}$, the linear sweep of $\lambda/\lambda_{\rm c}$ and the restriction of the driving strength $\Omega/2\pi \le 6$ MHz, the leakage to the $|2\rangle$ and higher excited states for each qubit can be restricted to, on average, within 3% for $\Delta_{\rm r}/2\pi = -30$ MHz, as experimentally monitored (Figures 3b and 3c in the main text. Data shown also reflect the readout uncertainty, $\sim 1\%$). State leakage is slightly higher for $\Delta_{\rm r}/2\pi = -20$ MHz, but in most experimental combinations of τ and Ω it is still less than 5% on average. For the small-percentage state leakage, Supplementary Equation 3 involving two-level qubits is a qualified model to describe the experimental situation.

By taking the next higher excited state $|2\rangle$ into consideration, we can model the experimental situation more accurately, as in the experiment we do not differentiate between P_1 and $P_{n\geq 1}$ when calculating $\langle J_z\rangle$. By including three energy levels for the qubit circuit, i.e., a qutrit, we compare the numerically-calculated (the thick black line) leakage-state probability $P_{\rm L}$ ($= P_2$ in the qutrit model) with the experimentally obtained values (thin lines) for the case of $\Delta_{\rm r}/2\pi = -30$ MHz, $\Omega/2\pi = 4$ MHz and $\tau = 600$ ns (Supplementary Figure 4a); in Supplementary Figure 4b, we obtain a fairly good agreement between numerical simulation (the dashed line) and the measured $\langle J_z\rangle$ curve.

Accuracy in tuning and synchronizing all four qubits' trajectory $\omega_{\rm q}(t)$: in the experiment to vary $\lambda/\lambda_{\rm c}$ linearly from 0.5 to 2.5 within the period of τ, i.e.,

$$\frac{\lambda}{\lambda_{\rm c}(t)} = 0.5 + \frac{2t}{\tau}, \text{ where } 0 \le t \le \tau, \tag{8}$$

we adjust the bias of each qubit such that the detuning of each qubit changes according to

$$\Delta_{\rm q}(t) = \frac{\lambda^2}{(0.5 + 2t/\tau)^2 \Delta_{\rm r}}. \tag{9}$$

Note that we cannot use the spectroscopy method to determine the qubit frequency during the steering of $\omega_{\rm q}(t)$ as the experimental process happens quickly in short times from a few to a few hundreds nanoseconds. Instead, we use a Ramsey-interference-like measurement to verify the accuracy in tuning $\omega_{\rm q}(t)$ by checking the dynamic phase accumulated during the process. Starting with the qubit frequency at $\omega_{\rm q}(0)$, we prepare the initial state $(|0\rangle - i|1\rangle)/\sqrt{2}$, and then tune the qubit frequency according to Supplementary Equation 9 for a time t, following which we apply a $\pi/2$ pulse with an adjusted phase

$$\theta = -\int_0^t \left[\omega_{\rm q}(t_1) - \omega_{\rm q}(0)\right] dt_1 = \frac{\lambda^2}{2\Delta_{\rm r}(0.5 + 2t/\tau)} - \frac{\lambda^2}{\Delta_{\rm r}} - \left[\omega_{\rm d} - \omega_{\rm q}(0)\right] t. \tag{10}$$

If the steering of ω_q is as expected, θ compensates the accumulated dynamic phase and we end up with measuring the envelope of the Ramsey interference fringe. The process is equivalent to the Ramsey interference measurement using two identical $\pi/2$ rotation pulses (Supplementary Figure 5a).

We note that this method has its limitations. First, due to the relatively short T_1 and T_2 of phase qubits, the probability of finding the qubit in $|1\rangle$, P_1, gradually decays as t increases, which renders the signal rather weak at larger t values. Second, this method cannot work when the qubit is tuned close to the resonator frequency, due to the Stark shift. As such, we test the accuracy of tuning the qubit frequency in a frequency range from 5.87 GHz to 6.14 GHz in 75 ns. The results for all four qubits are as expected (Supplementary Figure 5b).

Flux bias crosstalk from one qubit to the other is intended to be corrected during the steering of $\omega_q(t)$. However, we have no conclusive evidence that our synchronized steering of all four qubits is accurate and adequate, nor can we be absolutely sure about the accuracy of correcting the flux bias crosstalk. We estimate an uncertainty of $\delta\left[\omega_q(t)/2\pi\right] \approx 1$ MHz, based on all available experimental data and our knowledge of the control accuracy. In particular, the fact that most experimental data measured at the microwave drive strength $\Omega/2\pi = 2$ MHz show much lower $\langle J_z \rangle$ signals than expected is consistent with the estimated uncertainty level of $\omega_q(t)$. For this reason we choose intermediate microwave drive strengths $\Omega/2\pi = 4$ MHz and 6 MHz in the experiment.

Fortunately, the experimental impact of uncertainties in $\omega_q(t)$ can be greatly reduced by using larger negative detunings of Δ_r (for $\Delta_q(t)$ to sweep through smaller ranges in frequency, which also lowers the crosstalk impact) and longer sweep durations of τ. Using larger negative detunings of Δ_r also helps with minimizing state leakage. Therefore the experimental data agree quite well with numerical simulation that assumes perfect synchronized steering of $\omega_q(t)$ for most experimental combinations of Δ_r and τ. A slightly large disagreement is seen in the case of $\Delta_r/2\pi = -20$ MHz and $\tau = 600$ ns, which is compatible with our hypothesis of errors in synchronized tuning of $\omega_q(t)$.

Two-qubit behaviour and the off-resonant Ω: as a preparation for the four-qubit $\langle J_z \rangle$ measurements, we quickly perform the two-qubit measurements with Q_1-Q_2 and Q_1-Q_3, respectively, using pulse sequences that are similar to that shown in Figure 1c of the main text for the resonator and two of the qubits. It is observed that the two-qubit $\langle J_z \rangle$ curves also show the signature rise when λ/λ_c increases above 1. The two-qubit $\langle J_z \rangle$ measurements also confirm that Ω calibrated on-resonantly is applicable off-resonantly for small detunings of Δ_r (Supplementary Figure 6).

Supplementary References

[1] Zou, J. H., Liu, T., Feng, M., Yang, W. L., Chen C. Y. and Twamley, J. Quantum phase transition in a driven Tavis-Cummings model. *New J. Phys.* **15**, 123032 (2013).

[2] Rzaźewski, K., Wódkiewicz, K. and Źacowicz, W. Phase transitions, two-level atoms, and the A^2 term. *Phys. Rev. Lett.* **35**, 432-434 (1975).

[3] Bialynnicki-Birula, I. and Rzaźewski, K. No-go theorem concerning the superradiant phase transition in atomic systems. *Phys. Rev. A* **19**, 301-303 (1979).

[4] Nataf, B. and Ciuti, C. No-go theorem for superradiant quantum phase transitions in cavity QED and counter-example in circuit QED. *Nat. Commun.* **1**, 72 (2010).

[5] Vukics, A., Grießer, T. and Domokos, P. Elimination of the A-square problem from cavity QED. *Phys. Rev. Lett.* **112**, 073601 (2014).

[6] Milburn, G. J. and Alsing, P. Quantum phase transitions in a linear ion trap. *Dan Walls Memorial Volume*, edited by Carmichael, H., Glauber, R. and Scully, M. (Springer 2000); also available at LANL eprint quantum-ph/0003001v1, 2000.

[7] Pérez-Fernández, P., Relaño, A., Arias, J. M., Cejnar, P., Dukelsky, J. and García-Ramos, J. E. Excited-state phase transition and onset of chaos in quantum optical models. *Phys. Rev. E* **83**, 046208 (2011).

[8] Bastarrachea-Magnani, M. A., Lerma-Hernndez, S. and Hirsch, J. G. Comparative quantum and semiclassical analysis of atom-field systems: I. Density of states and excited-state quantum phase transitions, *Phys. Rev. A* **89**, 032101 (2014).

[9] Bastarrachea-Magnani, M. A., Lerma-Hernndez, S. and Hirsch, J. G. Comparative quantum and semiclassical analysis of atom-field systems: II. Chaos and regularity. *Phys. Rev. A* **89**, 032102 (2014).

[10] Martinis, J. M. Superconducting Phase Qubits. *Quantum Information Processing* **8**, 81-103 (2009).

[11] Wang, H. *et al.* Deterministic entanglement of photons in two superconducting microwave resonators. *Phys. Rev. Lett.* **106**, 060401 (2011).
